# Laparoscopically Harvested Pedicled Omental Flap in Immediate Unilateral Breast Reconstruction: A Systematic Review of Surgical Techniques and Clinical Outcomes

**DOI:** 10.3390/curroncol33070410

**Published:** 2026-07-09

**Authors:** Annie M. Wu, Surabi Thirugnanasampanthar, Muriel Brackstone

**Affiliations:** 1 General Surgery Residency Program, Western University Schulich Medicine & Dentistry, London, ON N6A 5A5, Canada; mwu268@uwo.ca; 2 Health Sciences Addition Building, Western University Schulich Medicine & Dentistry, London, ON N6A 5C1, Canada; sthirugnanasampanthar2028@meds.uwo.ca; 3 Division of General Surgery, London Health Sciences Centre, London, ON N6A 5A5, Canada

**Keywords:** breast reconstruction, omental flap, oncoplastic surgery, laparoscopic surgery, pedicled flaps, breast cancer

## Abstract

Laparoscopically harvested pedicled omental flap (LHPOF) reconstruction is a minimally invasive autologous option for immediate breast reconstruction, but prior reviews have largely examined omental flaps broadly, combining open and laparoscopic harvests, free and pedicled transfers, and mixed reconstructive indications. This systematic review evaluated operative characteristics, peri-operative complications, and esthetic outcomes following LHPOF after oncologic breast surgery. MEDLINE, Cochrane and Embase were searched without language or date restrictions, with backward citation searching of included studies. Eligible studies included female patients undergoing immediate unilateral breast reconstruction with LHPOF after mastectomy or BCS. Twenty-two studies published between 2001 and 2025 were included, representing 1869 patients. Operative techniques were broadly consistent, most commonly involving transverse-colon-first omental harvest, preservation of the right gastroepiploic vessels, and tunnelling from the inframammary fold toward the xiphoid. Reported complications included omental fat necrosis, partial flap loss, hematoma, infection, epigastric bulging, and incisional or tunnel-site hernia. Esthetic outcomes were generally favourable but assessed using heterogeneous methods. Current evidence suggests this technique is feasible in selected patients and may offer favourable esthetic outcomes with limited donor-site morbidity. However, prospective comparative studies with standardized reporting are needed to define optimal patient selection, long-term safety, and esthetic durability.

## Simple Summary

Breast reconstruction after cancer surgery can improve quality of life, but many reconstructive options involve additional scars, longer recovery, or donor-site complications. The omentum is a soft fatty tissue inside the abdomen that can be moved to the breast using laparoscopic surgery to help restore breast shape. In this review, we looked at published studies on the use of omental flaps in breast reconstruction to understand how the operation is performed, how safe it is, and what esthetic outcomes have been reported. The available studies suggest that this technique can be used in appropriately selected patients and may provide favourable breast shape and patient satisfaction, with relatively few abdominal donor-site complications. However, most studies were small and did not directly compare this operation with other reconstruction methods. More research is needed to better define which patients are most likely to benefit and how this technique compares with other options.

## 1. Introduction

Breast cancer is the most commonly diagnosed cancer among women in North America, with approximately one in eight women expected to develop breast cancer during their lifetime [1,2]. As part of standard treatment, many patients undergo mastectomy or BCS. Immediate autologous breast reconstruction has an established role in improving quality of life and psychological well-being following mastectomy [3]. Among available reconstructive options, LHPOF has emerged as a promising minimally invasive autologous technique, offering potential advantages such as minimal donor-site morbidity and favourable esthetic outcomes [4,5,6].

Despite growing interest, the current evidence base is largely derived from small, single-centre case series with inconsistent reporting of surgical technique, intraoperative parameters, complications, and esthetic outcomes [5,6]. Existing systematic reviews have often evaluated omental flaps broadly, combining open and laparoscopic harvests, pedicled and free transfers, and a range of reconstructive indications [7,8]. This heterogeneity limits the ability to accurately characterize operative approaches and outcomes specific to LHPOF in immediate unilateral breast reconstruction. Therefore, this review focuses specifically on laparoscopically harvested pedicled omental flaps for immediate unilateral breast reconstruction after mastectomy or breast-conserving surgery (BCS). By synthesizing operative technique, peri-operative complications, reconstructive outcomes, and esthetic results, we aim to outline common technical steps, examine factors relevant to patient selection, and identify outcomes that would benefit from more standardized reporting in future studies.

## 2. Materials and Methods

### 2.1. Study Design

This systematic review was conducted following the Preferred Reporting Items for Systematic Reviews and Meta-Analyses (PRISMA) 2020 guidelines [9]. The completed PRISMA checklist is provided in the Supplementary Materials [Table S1]. The review was registered with the PROSPERO registry of systematic reviews (CRD420251245375).

### 2.2. Eligibility Criteria

Studies were eligible for inclusion if they involved female patients with breast cancer undergoing immediate unilateral breast reconstruction with a laparoscopically harvested pedicled omental flap following mastectomy or BCS. No age restrictions were applied. Studies exclusively involving male patients were excluded. Eligible interventions included laparoscopically harvested pedicled omental flap reconstruction. Studies were excluded if outcomes for laparoscopic versus open omental harvest could not be clearly distinguished, or if outcomes for pedicled versus free omental flap reconstruction could not be separated. Both randomized and non-randomized study designs were considered eligible, including randomized controlled trials, observational cohort studies, case–control studies, cross-sectional studies, case series, and case reports. Reviews, meta-analyses, editorials, commentaries, animal studies, and in vitro studies were excluded.

### 2.3. Search Strategy

The search strategy was developed before study screening with input from a health sciences librarian. Searches were run in Ovid MEDLINE, Embase, and EBM Reviews-Cochrane Central Register of Controlled Trials, without restrictions on language or publication date. The strategy was adapted for each database and included relevant subject headings, including MeSH and Emtree terms, as well as keywords for breast reconstruction, omental flap reconstruction, laparoscopic harvest, and pedicled flap transfer. Backward citation searching was performed by reviewing the reference lists of included studies for additional relevant articles. The full search strategy is provided in Appendix A. No protocol amendments were made after PROSPERO registration.

### 2.4. Study Selection

**Figure 1:**
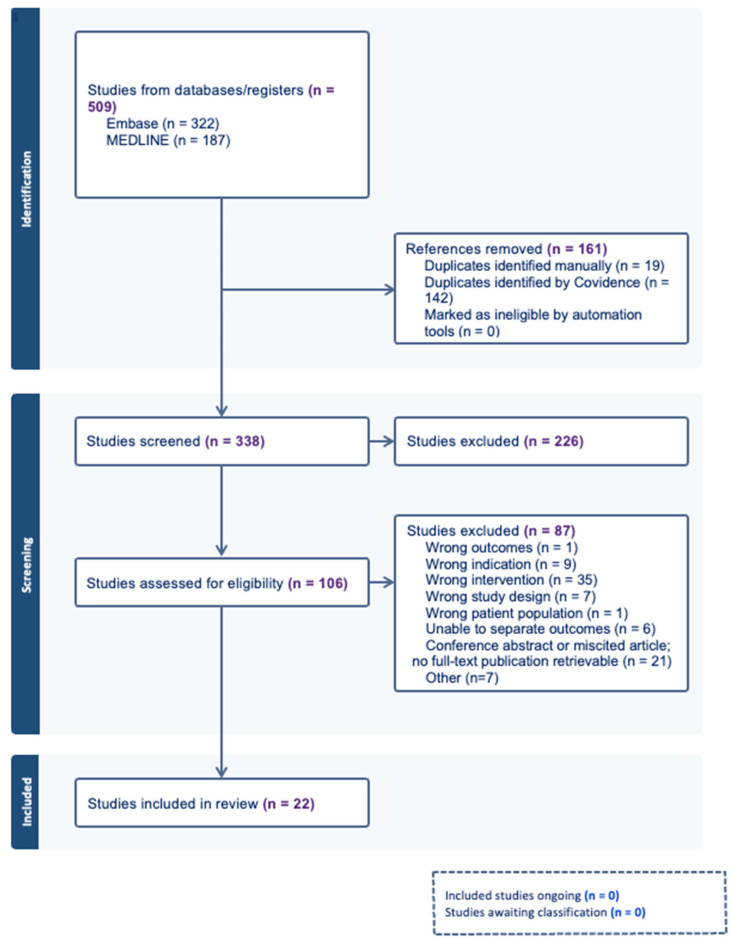
PRISMA Diagram of Study Screening and Selection; numbers presented in purple represent total numbers in that section and numbers presented in blue represent subtotal numbers within that section; n = number.

All records identified through the search were imported into a reference manager, and duplicates were removed before screening. Two reviewers independently screened titles and abstracts using the eligibility criteria. Articles considered potentially relevant underwent full-text review by both reviewers. Disagreements were resolved by discussion between the two reviewers. The study selection process is summarized in Figure 1.

### 2.5. Data Extraction

Data extraction was performed using a predefined form developed before full-text review. Both reviewers independently extracted data, and discrepancies were reviewed and resolved by consensus. Corresponding authors were contacted for clarification when methodological details were unclear or outcome data were missing.

Extracted variables included patient age, body mass index, breast cancer pathology and stage, medical comorbidities, smoking history, prior abdominal surgery, prior radiation therapy, and reported breast volume. As baseline characteristics were reported inconsistently across studies, the study characteristics section focused on variables most relevant to patient selection and operative planning.

Operative and technical characteristics were extracted as a primary domain and included patient and surgeon positioning, mastectomy incision type, laparoscopic port placement and configuration, pedicle approach and preservation, pedicle tunnelling route, hemostatic device, and flap fixation technique.

Clinical outcomes were also extracted as a primary domain, which included perioperative complications such as flap viability and success, total or partial flap necrosis, intraoperative vessel injury, postoperative infection, hematoma, fat necrosis, and donor-site morbidity, including infection, incisional hernia, abdominal wall bulging, and dehiscence.

Reconstructive and esthetic outcomes were extracted as a third primary domain and included patient-reported satisfaction and physician-reported or objective esthetic assessment.

Secondary outcomes included omental flap harvest time, total operative time, postoperative length of stay, oncologic follow-up duration, and local, regional, and distant recurrence.

### 2.6. Critical Appraisal

Methodological quality and risk of bias were assessed using the appropriate Joanna Briggs Institute (JBI) critical appraisal checklist according to study design [10,11]. Appraisal was used to inform interpretation of the evidence rather than to exclude studies solely on the basis of quality.

### 2.7. Data Synthesis

Given the anticipated heterogeneity in study design, operative technique, outcome definitions, follow-up duration, and reporting of complications and esthetic outcomes, a meta-analysis was not performed. Findings were synthesized descriptively and narratively. Outcomes were summarized using study-level proportions, reported ranges, and qualitative descriptions where appropriate. Primary outcomes were presented in tables and summarized in relation to consistency of reporting, methodological quality, and clinical relevance.

## 3. Results

### 3.1. Search Results

The search strategy and manual review of the literature identified a total of 509 studies for review, from which 161 duplicates were removed. A total of 338 studies were screened, yielding 106 studies for full-text review. 22 studies were included in the final systematic review (Figure 1).

### 3.2. Study Characteristics

All primary research studies included in the analysis were case series or observational cohort studies. There were no randomized controlled trials or case reports. Studies were published between 2001 and 2025, with larger cohorts emerging more recently. Although early reports generally consisted of small case series, later studies included larger retrospective cohorts, with sample sizes ranging from 5 to 300 patients. Overall, the 22 included studies had a median sample size of 58 patients and collectively represented 1869 patients undergoing LHPOF breast reconstruction (Table 1). Study periods, institutions, and cohort characteristics were reviewed to assess for potential overlap, and corresponding authors were contacted for clarification where overlap was uncertain. As partial cohort overlap could not be fully excluded in some reports, this number should be interpreted as the total reported sample across studies rather than a confirmed number of unique patients.

Reconstruction was most commonly performed following mastectomy. Several studies included BCS, either exclusively or within mixed cohorts. Where reported, patient age generally ranged from the late 30s to early 50s, while BMI was inconsistently described but typically fell within the normal to overweight range. Prior abdominal surgery was variably reported, with some studies describing low rates of prior cesarean section, laparoscopic cholecystectomy, appendectomy, or hysterectomy/oophorectomy, and others excluding major upper abdominal surgery. Other baseline characteristics, including smoking history, comorbidities, tumour pathology and stage, prior radiation therapy, and breast volume, were inconsistently reported across studies and were therefore not uniformly summarized. Overall, heterogeneity in study design, surgical indication, baseline reporting, and patient selection supported a descriptive narrative synthesis.

Critical appraisal of included studies using the JBI critical appraisal checklists indicated that most studies had low-to-moderate or moderate methodological concerns (Table 2 and Table 3). Common limitations included unclear consecutive or complete inclusion of participants, small sample sizes, incomplete baseline demographic or clinical reporting, variable follow-up duration, and limited use of validated patient-reported outcome measures.

### 3.3. Primary Outcomes

#### 3.3.1. Operative and Technical Characteristics

Operative and technical characteristics are summarized in Table 4. Across studies, the overall operative protocol and sequence were relatively consistent, with most describing a sequential approach in which oncologic breast resection was followed by LHPOF reconstruction. For omental flap harvest, multi-port laparoscopic approaches were more commonly used than single-port techniques, although exact port placement was inconsistently reported. Most studies described harvest using a transverse-colon-first dissection and preservation of the right gastroepiploic vessels. Subcutaneous tunnelling was most commonly performed from the medial inframammary fold toward the xiphoid process or linea alba. Overall, operative and technical descriptions were relatively consistent across studies, with only minor variation in subcutaneous tunnelling and omental flap fixation within the breast pocket.

Because omental volume can be difficult to predict before laparoscopic harvest and may be insufficient for total breast reconstruction, implant use was summarized separately as a reconstructive volume strategy rather than as an intraoperative complication (Table 5). Implant use with the omental flap was described in 9 of 22 papers overall. Among 12 mastectomy-only papers, 8 described addition of an implant with LHPOF, with reported rates ranging from 13% to 83%. In contrast, none of the 4 BCS-only papers described implant addition. Among 6 mixed mastectomy/BCS papers, 1 described implant use, with a reported rate of 12.6%.

#### 3.3.2. Peri-Operative Complications

Peri-operative complications are summarized in Table 6. Reporting of intraoperative complications was inconsistent across studies. Among reported intraoperative events, pedicle or vascular injury was the most frequently described complication across studies, although the absolute number of affected patients was low overall. In studies that reported management details, eight vascular pedicle compromise events were described. The omental flap was preserved in three cases (3/8, 37.5%), including two cases in which no change to the planned reconstruction was required because perfusion remained adequate, and one case in which hemostasis was achieved but partial flap volume loss occurred. In the remaining five cases (5/8, 62.5%), the flap was aborted or reconstruction was converted to an alternative approach, including implant-based reconstruction (2/8, 25.0%), free flap reconstruction (1/8, 12.5%), latissimus dorsi flap reconstruction (1/8, 12.5%), or omental flap removal (1/8, 12.5%). Failure to retrieve the omental flap was uncommon and was attributed to severe abdominal adhesions or insufficient omental volume. Visceral injury was rarely reported, and no study described conversion to laparotomy.

Postoperative reconstruction-site complications included omental fat necrosis, partial flap necrosis or volume loss, hematoma, infection, seroma, skin or nipple–areolar necrosis, transient omental firmness or induration, and less commonly flap displacement or bulging at the inframammary fold or subcutaneous tunnel. Most minor complications were managed conservatively or with drainage, aspiration, antibiotics, or local wound care, although some cases required operative evacuation, debridement, flap resection, or revision surgery. Several studies described postoperative firmness, swelling, or hardening of the omental flap as temporary, with gradual softening over weeks to months [13,25,30]. Omental fat necrosis was variably reported and ranged from conservative management to operative intervention; for example, El-Sherpiny et al. [14] reported post-radiation fat necrosis in 8 of 24 patients.

Donor-site complications included transient epigastric discomfort, fullness, epigastric or tunnel-site bulging, incisional, ventral, tunnel-site, or umbilical hernia, umbilical wound infection, and intra-abdominal infection. Most epigastric symptoms were self-limited and managed conservatively. Hernia was the most frequently reported donor-site complication and was often managed with surgical repair, although some cases were asymptomatic or treated non-operatively. Epigastric bulging was reported in several studies, including Kim et al. [19], who described epigastric bulging in 21.7% of patients, with spontaneous improvement in some cases over follow-up.

#### 3.3.3. Esthetic Outcomes

Esthetic outcomes are summarized in Table 7. Patient-reported satisfaction was described in 13 of 22 studies and was generally high, with reported satisfaction rates ranging from approximately 80% to 100%. However, assessment methods varied substantially, ranging from narrative satisfaction reporting and study-specific rating scales to validated assessment using BREAST-Q in one study. Physician-reported or objective esthetic outcomes were reported in 16 studies and were also favourable, with most studies reporting excellent or good outcomes in approximately 75% to 100% of patients. These outcomes were assessed using heterogeneous methods, including physician panel evaluation, Harris scale, BCCT.core software, S-BEST, and non-standard four-point cosmetic scales. Overall, available patient-reported and clinician/objective assessments suggested favourable esthetic outcomes following LHPOF reconstruction, although comparisons across studies were limited by inconsistent outcome measures.

### 3.4. Secondary Outcomes

#### 3.4.1. Operative Time and Postoperative Length of Stay

Intraoperative omental flap harvest time was reported in 12 studies, with reported values ranging from 40 to 180 min and a study-level median of approximately 66 min. Most studies reported mean harvest times between 40 and 81 min, while one early study reported improvement from 180 to 90 min with use of a harmonic scalpel [13].

Total operative time was reported in 16 studies, with reported values ranging from 112.5 to 510 min and a study-level median of approximately 225 min. When reported ranges were included, the overall reported range was 105 to 665 min. Operative time was variably reported and likely reflected differences in the extent of oncologic breast resection, addition of an implant, omental flap harvest and reconstruction technique, and institutional workflow.

Postoperative length of stay was reported in 15 studies, with reported values ranging from 2 to 15.3 days and a study-level median of approximately 7.5 days. When reported ranges were included, the overall reported range was 1 to 17 days. Length of stay was difficult to compare across studies because it likely reflected differences in institutional discharge practices. For example, one study report that longer postoperative admission reflected local practice, as patients were routinely discharged after completion of postoperative care, including suture removal [20].

#### 3.4.2. Oncologic Follow-Up and Recurrence Outcomes

Oncologic follow-up and recurrence outcomes are summarized in Table 8. Follow-up reporting varied across studies, with some reporting mean or median follow-up and others reporting ranges, interquartile ranges, or fixed follow-up intervals. Fixed follow-up values ranged from 8 to 90 months, and the overall reported follow-up range across all studies was 3 to 174 months. Four studies did not report follow-up duration.

Local recurrence was reported in 15 of 22 studies, with rates ranging from 0% to 12.5%. When reported, local recurrence sites most commonly involved the skin flap, nipple–areolar complex, papilla, or another breast quadrant after BCS. Regional recurrence was reported separately in two studies, with rates of 1.6% and 2.1%. Distant recurrence was reported in 12 studies, with rates ranging from 0% to 8.3%. Differences in follow-up duration and recurrence-site reporting limited direct comparison across studies.

## 4. Discussion

This systematic review supports LHPOF as a feasible reconstructive option for selected patients undergoing immediate breast reconstruction. Across studies, the operative sequence was generally consistent, with most studies preserving the right gastroepiploic vessels and describing omental dissection beginning at the transverse colon. Although technical details varied, including port number, tunnel location, and flap fixation, these shared steps suggest that LHPOF follows a consistent technical approach that could be standardized into a reproducible operative protocol. Several modifications were also described, including omental flap fixation to the chest wall and laparoscopic tunnel closure, which may reduce pedicle tension or kinking, improve flap positioning, and reflect ongoing efforts to optimize the procedure and reduce pedicle-related complications [12,15,18,19].

Patient selection appears central to the role of LHPOF reconstruction. Several studies described its use after mastectomy, while others used the omental flap for partial breast reconstruction after BCS. In the BCS setting, LHPOF may be particularly useful when standard glandular rearrangement would be insufficient, such as in small-breasted patients, larger anticipated resection volumes, or medially located defects that are difficult to reconstruct with conventional local tissue rearrangement. El-Sherpiny et al. [14] suggested that omental reconstruction may be advantageous in small breasts where local flaps risk distortion, while Zaha et al. [28] and Kim et al. [19] emphasized its utility for medial quadrant defects, including the upper medial “no man’s land” of the breast. However, in partial breast reconstruction, margin status remains an important consideration, and studies that incorporated intraoperative frozen section assessment highlight the need to ensure oncologic clearance before definitive reconstruction [16,30].

Complication profiles were generally acceptable, but several recurring issues have implications for technical refinement. Omental fat necrosis, partial flap loss, hematoma, infection, and transient flap firmness were reported across studies. Importantly, several authors described postoperative omental flap firmness as temporary, with gradual softening over weeks to months, which may be relevant for patient counselling and postoperative surveillance [13,25,30]. Donor-site morbidity was generally limited, but epigastric or tunnel-site bulging emerged as a notable technical consideration. Kim et al. [19] reported epigastric bulging in more than 20% of patients, many of which improved over time, and subsequently modified their technique by trimming subcutaneous fat over the tunnel to reduce tunnel thickness. This suggests that tunnel design, pedicle bulk, and closure technique may influence donor-site contour outcomes and should be considered in future technical standardization. Intraoperative perfusion assessment using ICG or near-infrared imaging may also help reduce flap-related complications, although current evidence remains limited [22].

Esthetic outcomes were generally favourable across both patient-reported and clinician/objective assessments, although outcome measurement was heterogeneous. Studies used a range of tools, including narrative satisfaction, non-standard cosmetic scales, physician panel assessment, Harris scale, BCCT.core, S-BEST, and BREAST-Q. Fabrizio et al. [15] provided one of the few validated patient-reported assessments, reporting improvement in BREAST-Q domains between 6 months and 1 year, including satisfaction with breast appearance and psychosocial, sexual, and physical well-being. Several studies also reported preserved contour or volume after radiotherapy, although radiotherapy-specific outcomes were not consistently stratified and should be interpreted cautiously [4,21,22,25]. Together, these findings suggest that LHPOF may achieve favourable esthetic results in appropriately selected patients, but standardized patient-reported and objective esthetic outcomes are needed to better define its comparative value.

The oncologic safety of LHPOF in breast reconstruction is an important consideration, particularly given the theoretical concern that adipose-rich tissue and the angiogenic properties of the omentum could support tumour growth. Ni et al. highlighted this concern, noting that adipocytes may promote breast cancer cell growth through local estrogen production, adipocytokine signalling, and the high concentration of angiogenic stem cells within the omentum [7]. However, available clinical data have not demonstrated a clear increase in recurrence risk with LHPOF reconstruction. In the studies included in this review, recurrence rates were generally low. Local recurrence ranged from 0% to 12.5%, although the highest rate came from a small series, and larger studies more commonly reported rates between 0.7% and 3.2%. Distant recurrence ranged from 0% to 8.3%. Reassuringly, several larger cohort series provided both meaningful sample sizes and longer follow-up, including cohorts of 129 to 300 patients and follow-up extending to 90 months or, in one study, up to 12 years [4,5,19].

These rates appear comparable to recurrence rates reported after standard BCS or immediate breast reconstruction, as a 5.3% ipsilateral breast tumour recurrence prevalence was reported in a large meta-analysis of BCS [31]. Similarly, a systematic review of stage I–II breast cancer patients undergoing mastectomy reported local recurrence rates of 3.2% after mastectomy with immediate breast reconstruction and 2.1% after mastectomy alone, with no increased risks after adjustment for age and follow-up time [32]. In the current review, most reported local recurrences involved the reconstructed breast, skin flap, nipple–areolar complex, papilla, or another breast quadrant, rather than clearly involving the omental flap itself. However, recurrence site was not consistently reported, and the available evidence remains limited by retrospective study design, heterogeneous indications, and variable follow-up duration. Taken together, current evidence does not suggest that omental flap reconstruction compromises oncologic safety; however, this conclusion should be interpreted cautiously given the predominance of retrospective, non-comparative studies and inconsistent recurrence reporting.

Within the contemporary reconstructive algorithm, LHPOF is best viewed as an additional autologous option alongside established techniques such as deep inferior epigastric perforator (DIEP) flap reconstruction. Most included studies were non-comparative case series, and direct comparisons between LHPOF and other reconstructive techniques remain limited. LHPOF for breast reconstruction post mastectomy may be more suitable for selected patients with small-to-moderate breast volume, limited abdominal subcutaneous tissue, or a preference to avoid the donor-site morbidity associated with DIEP. The study by Yoon et al. provides, to our knowledge, one of the few comparative analyses, evaluating 236 patients who underwent single-port LHPOF reconstruction after nipple-sparing mastectomy or BCS and comparing cosmetic outcomes with a matched DIEP flap cohort [27]. Cosmetic satisfaction rates were similar between the omental flap and DIEP groups (82.5% vs. 76.4%, *p* = 0.467), and the authors reported that cosmetic outcomes after omental flap reconstruction were not inferior to those after DIEP reconstruction in appropriately selected patients. Notably, the assessment was limited to images of the reconstructed breast and did not account for abdominal donor-site appearance. Given the substantial difference in abdominal scarring between DIEP and LHPOF procedures, inclusion of donor-site appearance may further favour LHPOF in eligible patients while achieving comparable breast cosmesis. Further comparative studies against established autologous options such as DIEP flap reconstruction are also needed, but should include both breast esthetic outcomes and abdominal donor-site morbidity to provide a balanced assessment of reconstructive trade-offs [27].

The main limitations of the current evidence are the predominance of small retrospective case series, inconsistent reporting of baseline characteristics, variable follow-up, and limited comparative data. Potential cohort overlap was also a limitation. Study dates, institutions, and cohort characteristics were reviewed to identify possible overlap, and corresponding authors were contacted for clarification where uncertainty remained. Studies with evidently duplicative cohorts were excluded where identifiable. However, possible but unconfirmed partial overlap could not be fully excluded in some reports. Important outcomes such as donor-site morbidity, abdominal symptoms, radiotherapy effects, capsular contracture in combined implant cases, recurrence, and patient-reported satisfaction were not uniformly reported. Preoperative volume prediction also remains a key challenge, as insufficient omental volume frequently led to implant augmentation or alternative reconstruction. Future studies should evaluate standardized methods for estimating omental volume, including CT-based assessment or three-dimensional planning, and should clarify which patients are most likely to achieve adequate reconstruction with omentum alone versus combined omental flap–implant reconstruction [18,19,26,29].

Overall, LHPOF appears to be a promising minimally invasive autologous reconstruction option for selected patients. Despite the lower donor-site morbidity and favourable esthetic outcomes reported in this systematic review, the technique is not widely established and remains relatively unfamiliar in North America. Limited uptake may reflect surgeon-, centre-, and system-level factors, including lack of familiarity with the procedure, limited comparative safety and esthetic outcome data relative to established reconstructive options, difficulty predicting omental volume preoperatively, potential risk of intra-abdominal complications, and logistical concerns related to workflow and introduction of a new technique. Further work is needed to explore surgeon perspectives on the barriers, facilitators, feasibility, and appropriateness of implementing LHPOF in healthcare centres.

## 5. Conclusions

LHPOF reconstruction is a feasible minimally invasive autologous option for immediate unilateral breast reconstruction in selected patients, with generally favourable esthetic outcomes and limited major donor-site morbidity reported across the available literature. Major complications, such as complete flap loss, were uncommon, while commonly reported complications such as fat necrosis, hematoma, infection, and seroma are similar in type to those seen with other autologous reconstructive techniques. However, variation in operative technique, volume assessment, follow-up, and outcome reporting limits comparability across studies. Future prospective comparative studies are needed to standardize technique and reporting, clarify patient selection, and define the long-term safety, esthetic durability, and reconstructive role of LHPOF within contemporary breast reconstruction.

## Figures and Tables

**Table 1:** Study Characteristics.

Authors/Year of Publication	Country	Study Design ^1^	Number of Patients	Indications for Reconstruction ^2^	Mean Age	Mean BMI ^3^	History of Abdominal Surgeries
Byon, 2025 [12]	South Korea	CS	8	Mastectomy	38	NR	NR
Cothier-Savey, 2001 [13]	France	CS	10	Mastectomy	48	NR	None
El-Sherpiny, 2021 [14]	Egypt	Cohort	24	BCS	45	NR	NR; prior upper abdominal open surgery excluded
Fabrizio, 2022 [15]	Italy	CS	12	Mastectomy	49	29	NR
Guan, 2015 [16]	China	CS	25	BCS	43	NR	NR; upper abdominal surgery excluded
Hu, 2025 [17]	China	CS	90	Mastectomy	42	NR	NR; upper abdominal surgery/peritonitis excluded
Hu, 2024 [18]	China	Cohort	200	Mastectomy	42, median	22, median	NR; omentectomy and major upper abdominal surgery excluded
Kahter, 2023 [6]	Egypt	CS	95	Mastectomy	43	34	History of laparotomy: 9.5%
Kim, 2020 [19]	South Korea	CS	129	BCS 17.8%; Mastectomy 82.2%	45	NR	NR; upper abdominal laparotomy excluded
Kim, 2017 [20]	South Korea	CS	5	Mastectomy	44	23	NR; no history of multiple abdominal surgeries
Liu, 2024 [4]	China	CS	300	Mastectomy	41, median	23	C-Section 5.6%; laparoscopic cholecystectomy 4.0%; appendectomy 3.7%; hysterectomy/oophorectomy 1.0%; other 1.3%
Park, 2025 [21]	South Korea	Cohort	208	BCS 10.6%; Mastectomy 89.4%	47	23	NR; upper abdominal laparotomy excluded
Park, 2020 [22]	South Korea	CS	8	Mastectomy 75.0%	45	23	NR
Shen, 2024 [5]	China	CS	65	BCS 24.2%; Mastectomy 75.8%	NR	NR	NR
Shen, 2019 [23]	China	Cohort	53	Mastectomy	NR	NR	NR
Shen, 2021 [24]	China	CS	63	Mastectomy	NR	NR	NR; upper abdominal surgery/peritonitis excluded
Song, 2011 [25]	China	CS	5	BCS	42	NR	NR; no history of upper abdominal surgery/peritonitis
Wang, 2020 [26]	China	CS	10	Mastectomy	49	NR	30%
Yoon, 2025 [27]	South Korea	Cohort	236	BCS 9.7%; Mastectomy 90.3%	46	23	NR
Zaha, 2017 [28]	Japan	CS	190	BCS 77.0%; Mastectomy 23.0%	51	NR	NR; upper abdominal laparotomy excluded
Zhang, 2019 [29]	China	Cohort	93	BCS	40	22	C-section: 1.9%/4.6%; laparoscopic salpingectomy 1.9%/2.3%; laparoscopic appendectomy: 2.3%/3.8%
Zhang, 2015 [30]	China	CS	40	Mastectomy	39	23	C-Section 2.5%; laparoscopic salpingectomy 2.5%; laparoscopic appendectomy 2.5%

^1^ CS = case series; ^2^ BCS = breast-conserving surgery; NR = not reported,^3^ BMI = body mass index.

**Table 2:** Critical Appraisal of Study Quality and Risk of Bias using JBI for Case Series ^1^.

Author/Year	CS1	CS2	CS3	CS4	CS5	CS6	CS7	CS8	CS9	CS10	Overall Appraisal
Byon, 2025 [12]	N	Y	Y	U	U	Y	Y	Y	Y	Y	Moderate
Cothier-Savey, 2001 [13]	U	Y	Y	U	U	N	Y	Y	Y	Y	Moderate
Fabrizio, 2022 [15]	Y	Y	Y	U	U	Y	Y	Y	Y	Y	Moderate
Guan, 2015 [16]	Y	Y	Y	U	U	Y	Y	Y	Y	Y	Low-to-moderate
Hu, 2025 [17]	Y	Y	Y	U	U	Y	Y	Y	Y	Y	Moderate
Kahter, 2023 [6]	Y	Y	Y	U	U	Y	Y	Y	Y	Y	Low-to-moderate
Kim, 2020 [19]	Y	Y	Y	U	U	Y	Y	Y	Y	Y	Moderate
Kim, 2017 [20]	Y	Y	Y	U	U	Y	Y	Y	Y	Y	Low-to-moderate
Liu, 2024 [4]	Y	Y	Y	U	U	Y	Y	Y	Y	Y	Low-to-moderate
Park, 2020 [22]	Y	Y	Y	U	U	Y	Y	Y	Y	Y	Moderate
Shen, 2024 [5]	Y	Y	Y	U	U	Y	Y	Y	Y	Y	Moderate
Shen, 2021 [24]	Y	Y	Y	U	U	Y	Y	Y	Y	Y	Moderate
Song, 2011 [25]	Y	Y	Y	U	U	Y	Y	Y	Y	Y	Moderate
Wang, 2020 [26]	Y	Y	Y	U	U	Y	Y	Y	Y	Y	Low-to-moderate
Zaha, 2017 [28]	Y	Y	Y	Y	Y	Y	Y	Y	Y	Y	Low-to-moderate
Zhang, 2015 [30]	Y	Y	Y	U	U	Y	Y	Y	Y	Y	Low-to-moderate

^1^ Abbreviations: Y, yes; N, no; U, unclear. CS1 = clear inclusion criteria; CS2 = condition measured in a standard and reliable way; CS3 = valid methods used to identify the condition; CS4 = consecutive inclusion of participants; CS5 = complete inclusion of participants; CS6 = demographic characteristics clearly reported; CS7 = clinical information clearly reported; CS8 = outcomes and follow-up clearly reported; CS9 = site or clinic demographic information clearly reported; CS10 = statistical analysis appropriate.

**Table 3:** Critical Appraisal of Study Quality and Risk of Bias using JBI for Cohort Studies ^1^.

Authors	Year	C1	C2	C3	C4	C5	C6	C7	C8	C9	C10	C11	Overall Appraisal
El-Sherpiny, 2021 [14]	2021	Y	Y	Y	U	U	Y	Y	U	U	U	U	Moderate
Hu, 2024 [18]	2024	Y	Y	Y	U	U	Y	Y	U	U	U	U	Moderate
Park, 2025 [21]	2025	Y	Y	Y	U	U	Y	Y	Y	U	U	U	Low-to-moderate
Shen, 2019 [23]	2019	U	Y	Y	U	U	Y	Y	U	U	U	U	Moderate
Zhang, 2019 [29]	2019	Y	Y	Y	U	U	Y	Y	U	U	U	U	Moderate

^1^ Abbreviations: Y, yes; N, no; U, unclear. C1 = groups similar and recruited from same population; C2 = exposures measured similarly between groups; C3 = exposure measured validly and reliably; C4 = confounding factors identified; C5 = strategies used to address confounding; C6 = participants free of outcome at baseline; C7 = outcomes measured validly and reliably; C8 = follow-up time sufficient; C9 = follow-up complete; C10 = loss to follow-up described and managed; C11 = statistical analysis appropriate.

**Table 4:** Operative and Technical Characteristics.

	Studies Reported (n/N)	Reported Approaches	Key Findings/Notes
Operative sequence	22/22	Breast surgery followed by LHPOF (18); LHPOF before breast resection (3); concurrent mastectomy and omental harvest (1)	Breast resection followed by subcutaneous tunnelling and laparoscopic omental harvest was the most commonly described sequence.
Number of laparoscopic ports	16/22	Single-port approach (2); multi-port approach using 3–5 abdominal ports/trocars (14)	Most studies used an umbilical camera port with additional lateral abdominal working ports.
Pedicle approach	21/22	Transverse-colon-first dissection (19); stomach-first dissection (2)	Dissection from the transverse colon was the dominant approach, usually proceeding from the splenic toward hepatic flexure with entry into the lesser sac.
Pedicle preservation	20/22	Right gastroepiploic pedicle (19); affected-side gastroepiploic pedicle (1)	The right gastroepiploic artery/vein was preserved in nearly all studies that reported pedicle selection.
Subcutaneous tunnelling location	21/22	Medial inframammary fold to xiphoid/linea alba (13); IMF to subcostal/costal margin (3); other or unclear tunnel modifications (5)	A two-finger-width tunnel from the medial IMF toward the xiphoid/linea alba was commonly described.
Omental flap fixation	16/22	Sutured to pectoralis/chest wall/breast tissue/IMF (13); Ti-loop mesh suspension (1); no fixation required (1); flap transferred without detailed fixation (1)	Flap fixation technique was heterogeneous. Several studies fixed the pedicle near the tunnel opening or linea alba to reduce twisting or herniation.

IMF = inframammary fold; n/N = number of studies reporting the variable out of 22 included studies.

**Table 5:** Implant use in addition to LHPOF reconstruction by indication.

Reconstruction Indication	Studies (n/N)	Studies with Implant Addition	Reported Rate (%)
Mastectomy	12/22	8/12	13–83%
BCS	4/22	0/4	0%
Mixed mastectomy and BCS	6/22	1/6	12.6%
Overall	22/22	9/22	12.6–83%

BCS = breast-conserving surgery; n/N = number of papers among 22 included studies. Implant addition refers to implant placement described as part of the reconstructive procedure with the omental flap.

**Table 6:** Peri-Operative Complications.

	Studies Reporting (n/N) ^1^	Reported Range (%)	Intervention/Management
Intraoperative			
Pedicle or vascular injury			
- Flap Preserved	3/16	0.5–20	No change to the planned reconstruction when perfusion was adequate (2); Managed with hemostasis but resulted in partial flap volume loss (1)
- Flap Aborted or converted	5/16	0.3–1.1	Conversion to implant-based reconstruction (2), conversion to free flap (1), omental flap removal (1), or conversion to latissimus dorsi flap reconstruction (1)
Failure to retrieve omental flap	2/16	2.1–4.0	Converted to alternative reconstruction due to severe abdominal adhesions (1), insufficient omentum (2)
Visceral injury	1/16	1.1	Managed intraoperatively (1)
Conversion to laparotomy	0/16	0	
Postoperative—Reconstruction Site			
Omental flap firmness/nodules	5/21	3.4–10.0	Some were transiently firm and resolved spontaneously after a few months, while other studies reported no improvement
Omental fat necrosis	9/21	2.5–33.3	Usually managed conservatively; some cases required prolonged drainage, debridement, partial operative resection (4), complete omental flap removal (2)
Partial omental flap loss	2/21	2.0–5.0	Operative resection (2)
Complete omental flap loss	1/21	3.3	Operative resection (2)
Hematoma/hemorrhage	9/21	1.0–20.0	Conservative management, with some cases requiring drainage or operative evacuation (5)
Seroma	3/21	1.9–8.7	Prolonged drainage or repeated aspiration
Breast skin flap dehiscence	2/21	3.3–6.0	Conservative treatment with dressings or delayed secondary closure
Breast envelope necrosis	6/21	0.8–5.4	Usually managed conservatively and resolved spontaneously
Infection	5/21	1.0–7.6	Antibiotics, drainage, incision and drainage, dressing changes
Chylous Leakage	1/21	0.5	
Omental flap displacement	1/21	10.0	Operative Revision
Bulge of inframammary fold or subcutaneous tunnel	2/21	3.3–20.0	Operative removal of adipose tissue in subcutaneous tunnel (2)
Postoperative—Donor Site			
Epigastric discomfort	3/17	5.0–16.7	Transient and managed conservatively
Epigastric bulging	2/17	14.4–21.7	Majority resolved spontaneously
Ventral, incisional, tunnel, or umbilical hernia	9/17	0.3–14.0	Majority underwent hernia repair, often with mesh; some were managed conservatively or were asymptomatic
Umbilical wound infection	3/17	0.8–1.6	Conservative management and resolved spontaneously
Intra-abdominal infection	2/17	0.4–0.8	Percutaneous drainage and intravenous antibiotics

^1^ n/N = number of studies reporting at least one occurrence of the event/number of studies reporting data for that outcome. Values in parentheses indicate the total number of patient cases reported across those studies. Reported ranges reflect patient-level event rates within individual studies and were not pooled. At the section level, 6 studies did not report intraoperative event data, 1 study did not report postoperative reconstruction-site complication data, and 5 studies did not report donor-site complication data.

**Table 7:** Subjective and Objective Esthetic Outcomes.

Outcome	Studies Reported (n/N)	Reported Range (%)	Key Findings/Notes ^1^
Patient-reported satisfaction	13/22	80–100	Patient satisfaction was generally high across studies, although assessment methods were heterogeneous and often non-validated. Reported measures included narrative satisfaction, study-specific satisfaction scales, esthetic satisfaction ratings, and BREAST-Q in one study.
Physician- reported/ Objective assessment	16/22	75–100 excellent/good	Favourable esthetic outcomes were commonly reported using variable assessment methods, including physician panel review, Harris scale, BCCT.core, S-BEST, and non-standard four-point cosmetic scales.

^1^ BCCT.core = Breast Cancer Conservative Treatment cosmetic results software; S-BEST = Seoul Breast Esthetic Scoring Tool; n/N = number of studies reporting the outcome among 22 included studies.

**Table 8:** Oncologic Follow-up and Recurrence Outcomes.

Study	Follow-Up Duration, Months ^1^	Local Recurrence ^2^	Regional Recurrence	Distant Recurrence
Byon, 2025 [12]	24	1/8, 12.5%	NR	NR
Cothier-Savey, 2001 [13]	NR	NR	NR	NR
El-Sherpiny, 2021 [14]	Mean 18, (15–24)	NR	NR	NR
Fabrizio, 2022 [15]	24	NR	NR	NR
Guan, 2015 [16]	Mean 32, (6–51)	1/24, 4.2%	NR	2/24, 8.3%
Hu, 2025 [17]	16	1/90, 1.1%; papillary recurrence n = 1	NR	0/90, 0%
Hu, 2024 [18]	Median 16, (3–24)	NR	NR	NR
Kahter, 2023 [6]	Median 60	1/92, 1.1%	NR	3/92, 3.3%
Kim, 2020 [19]	Median 38	3/129, 2.3%; Paget’s disease n = 2, skin flap n = 1	2/129, 1.6%	3/129, 2.3%
Kim, 2017 [20]	Mean 8, (5–11)	0/5, 0%	NR	0/5, 0%
Liu, 2024 [4]	Median 32, (10–55)	2/300, 0.7%	NR	1/300, 0.3%
Park, 2025 [21]	Median 60, (IQR 42–84)	NR	NR	NR
Park, 2020 [22]	NR	NR	NR	NR
Shen, 2024 [5]	Mean 52 ± 38 ^3^	2/62, 3.2%	NR	1/62, 1.6%
Shen, 2019 [23]	NR	NR	NR	NR
Shen, 2021 [24]	NR	2/60, 3.3%	NR	NR
Song, 2011 [25]	Mean 8 (5–11)	0/5, 0%	NR	0/5, 0%
Wang, 2020 [26]	Mean 22 (15–28)	0/10, 0%	NR	0/10, 0%
Yoon, 2025 [27]	Median 59	7/236, 3.0%; Paget’s disease n = 4, skin flap n = 3	5/236, 2.1%	7/236, 3.0%
Zaha, 2017 [28]	Median 90, (5–174)	2/200, 1.0%; another quadrant after BCS n = 1, nipple after NSM n = 1	NR	NR
Zhang, 2019 [29]	6–30	0/93, 0%	NR	0/93, 0%
Zhang, 2015 [30]	Mean 16, (6–36)	0/40, 0%	NR	0/40, 0%
**Summary**	Fixed follow-up: 8–90 months (overall range 3–174 months)	Reported in 15/22 studies; 0–12.5%	Reported in 2/22 studies; 0–2.1%	Reported in 12/22 studies; 0–8.3%

^1^ Follow-up duration is reported in months according to the original study. Values are represented as mean, median, or range or fixed follow-up time, as reported. Ranges, interquartile range, and standard deviations are included when available. NR indicates not reported; ^2^ Recurrence outcomes are reported as n/N (%), where n represents the number of patients with recurrence and N represents the total number of patients in the study or reporting cohort. Percentages were calculated using the study denominator unless otherwise reported by the original authors; ^3^ Survival follow-up was available for 56/62 patients in the pedicled LHOF group (90.3%), as reported by the original study.

## Data Availability

The original contributions presented in this study are included in the article/Supplementary Materials. Further inquiries can be directed to the corresponding author.

## Supplementary Materials

The following supporting information can be downloaded at: https://www.mdpi.com/article/10.3390/curroncol33070410/s1, Table S1: PRISMA_2020_checklist.

## Abbreviations

The following abbreviations are used in this manuscript:

BCS	Breast Conserving Surgery
DIEP	Deep inferior epigastric perforator
JBI	Joanna Briggs Institute (JBI)
LHPOF	Laparoscopically harvested pedicled omental flap

## Appendix A

The full electronic search strategies are provided below. Results were imported into Covidence for screening under the review title Laparoscopically Harvested Pedicled Omental Flap in Immediate Unilateral Breast Reconstruction: A Systematic Review of Surgical Techniques and Clinical Outcomes.

**Table A1:** Ovid MEDLINE search strategy <1946 to 15 December 2025>.

Search Strategy	Results
exp Mastectomy/or mastectom$.mp. or (breast$ adj5 reconstruct$).tw,kf,kw.	60,064
Omentum/or (omentum$ or omental$).mp.	18,536
1 and 2	148
Free Tissue Flaps/or exp Surgical Flaps/	72,720
((free adj2 flap$1) or (free adj2 graft$)).tw,kf,kw.	24,695
free tissue$ transfer$.tw,kf,kw.	4114
((breast$ or tissue$) adj5 flap$1).tw,kf,kw.	12,511
(flap$1 adj3 (island or surgical)).tw,kf,kw.	6916
or/4–8	88,367
2 or (pedicl$ OF or pedicl$ LHO or LHPOFs or LHPOF or pedicl$).mp.	58,648
Laparoscopy/or Laparoscopes/or * Surgical Procedures, Minimally Invasive/or Robotics/or robot$.tw,kf,kw. or (mini$ invasive$ or laparoscop$ or celioscop$ or peritoneoscop$).tw,kf,kw.	373,975
1 and 9 and 10 and 11	81
3 or 12	187

In the search strategy, the asterisk (*) primarily functions as a truncation or wildcard symbol.

**Table A2:** Embase Classic and Embase search strategy <1947 to 12 December 2025>.

Search Strategy	Results
exp mastectomy/or breast reconstruction/or exp * breast tumour/su or mastectom$.tw,kf,kw. or (breast$ adj5 reconstruct$).tw,kf,kw.	136,781
exp omentum/or omental flap/or omental pedicle flap/or pedicled omentum graft/or (omentum$ or omental$).tw,kf,kw.	29,279
1 and 2	273
exp surgical flaps/or tissue flap/or exp skin flap/or pedicled skin flap/	58,230
((free adj2 flap$1) or (free adj2 graft$)).tw,kf,kw.	30,578
free tissue$ transfer$.tw,kf,kw.	4606
((breast$ or tissue$) adj5 flap$1).tw,kf,kw.	14,902
(flap$1 adj3 (island or surgical)).tw,kf,kw.	9030
or/4–8	82,473
2 or pedicled skin flap/or (pedicl$ OF or pedicl$ LHO or LHPOFs or LHPOF or pedicl$).tw,kf,kw.	86,307
exp laparoscopy/or laparoscope/or * minimally invasive surgery/or Robotics/or robot$.tw,kf,kw. or (mini$ invasive$ or laparoscop$ or celioscop$ or peritoneoscop$).tw,kf,kw.	597,520
1 and 9 and 10 and 11	101
3 or 12	322

In the search strategy, the asterisk (*) primarily functions as a truncation or wildcard symbol.

**Table A3:** EBM Reviews-Cochrane Central Register of Controlled Trials <November 2025>.

Search Strategy	Results
exp Mastectomy/or mastectom$.mp. or (breast$ adj5 reconstruct$).tw,kf,kw.	7759
Omentum/or (omentum$ or omental$).mp.	397
1 and 2 (0)	
Free Tissue Flaps/or exp Surgical Flaps/	1861
((free adj2 flap$1) or (free adj2 graft$)).tw,kf,kw.	1202
free tissue$ transfer$.tw,kf,kw.	67
((breast$ or tissue$) adj5 flap$1).tw,kf,kw.	867
(flap$1 adj3 (island or surgical)).tw,kf,kw.	463
or/4–8	3676
2 or (pedicl$ OF or pedicl$ LHO or LHPOFs or LHPOF or pedicl$).mp.	2148
Laparoscopy/or Laparoscopes/or * Surgical Procedures, Minimally Invasive/or Robotics/or robot$.tw,kf,kw. or (mini$ invasive$ or laparoscop$ or celioscop$ or peritoneoscop$).tw,kf,kw.	46,028
1 and 9 and 10 and 11	0
3 or 12	0

In the search strategy, the asterisk (*) primarily functions as a truncation or wildcard symbol.
